# Impact of inflammation on anemia of hemodialysis patients who were under treatment of recombinant human erythropoietin

**DOI:** 10.12861/jrip.2013.30

**Published:** 2013-09-01

**Authors:** Mahmoud Rafiean-Kopaie, Hamid Nasri

**Affiliations:** ^1^Medical Plants Research Center, Shahrekord University of Medical Sciences, Shahrekord, Iran; ^2^Department of Nephrology, Shahrekord University of Medical Sciences, Shahrekord, Iran

**Keywords:** End-stage Renal Disease, Ferritin, Hemodialysis, Anemia, Malnutrition, C-reactive Protein

## Abstract

The aim of this study was to investigate the association between serum C-reactive protein (CRP) as the marker of inflammation with serum ferritin level and its role in the severity of anemia of hemodialysis (HD) patients. A cross-sectional study was conducted on a group of stable hemodialysis patients. There were 36 patients overall, 25 of which were non-diabetic and 11 were diabetic mean patients’ age was 53 (±15.8) years. In this study, there was a significant inverse correlation between serum ferritin and hematocrit level (r=-0.34, p=0.044) (adjusted for age). Also a significant positive correlation between serum CRP and serum iron (r=0.44, p=0.008) (adjusted for age too) was observed. Moreover, in male HD patients, a significant positive correlation between serum CRP and serum ferritin (r=0.56, p=0.009) was found. Malnutrition and inflammation in hemodialysis may increase serum ferritin concentration apart from iron status, and needs more attention during iron therapy for the treatment of anemia in HD patients.

**Implication for health policy/practice/research/medical education:**

Malnutrition-inflammation complex syndrome, may increase serum ferritin concentration apart from iron status, and needs more attention during iron therapy for the treatment of anemia in regular hemodialysis patients.

## Introduction

Anemia in end-stage kidney disease can be managed by recombinant human erythropoietin (EPO) (1). Iron administration plays a central role in enhancing anemia responsiveness to EPO (2). Serum ferritin level and iron saturation ratio are the two most commonly used markers of iron status in maintenance hemodialysis patients (1-3). Inflammation is quite common in hemodialysis (HD) patients, and its prevalence between HD patients may be as high as 40 to 60% (3-5). Inflammation, is closely related to protein-energy malnutrition in dialysis patients and the simultaneous combination of these two conditions, also referred to as malnutrition-inflammation syndrome (2-5). It was shown that high levels of serum ferritin are engendered by inflammatory status apart from iron stores (1-4). Serum ferritin is also an acute phase reactant (3-8). While malnutrition-inflammation syndrome may play a central role in poor clinical outcome, including a high rate of mortality, diminished quality of life and hospitalization, it may also lead to high level of ferritin and refractory anemia including unresponsiveness to EPO in these patients (6-8). In fact, the erythropoiesis suppressing effect of inflammation is mainly due to an increased activity of the pro-inflammatory cytokines (5-8). *In vivo*, the cytokines act in concert with affect precursor cells at different stages of erythropoiesis. Cytokines, TNF-*alfa *and IL-1 have been studied (2-9). The inhibitory effect of TNF-*alfa *and IL-1 on erythropoiesis can be overcome in a dose-dependent fashion by administering EPO (2-9). It is thought that the direct inhibitory effect on erythroid precursors is primarily due to alterations in sensitivity to erythropoietin (2-9). C-reactive protein (CRP) is secreted by the liver and inflammation causes a rapid increase in its serum concentration. It plays a role in the host defense by interacting with the complement. Compared to measurements of other markers of inflammation and the acute-phase reaction, serum CRP has several advantages. It is a simple, reliable, readily available and inexpensive test. It is also a long-term predictor of cardiovascular risk and mortality in general population and in chronic kidney disease patients (4-9). It is thought that during the acute phase response, inflammatory cytokines such as interleukin 1 beta (IL-1ß) and tumor necrosis factor alpha (TNF-*alfa*) increase the synthesis of both H and L subunits of ferritin. Hence, serum ferritin can be elevated in inflammation as mentioned above (6-9).

## Objectives

We aimed to test the association between serum CRP and serum ferritin and its impact on the severity of anemia of regular HD patients, and to determine their association with malnutrition-inflammation complex syndrome.

## Patients and Methods

### Patients

This cross-sectional study was conducted on a group of end-stage renal disease patients, who underwent hemodialysis treatment The etiologies of renal failure were different, containing mainly hypertension, diabetic nephropathy, various glomerular diseases, autosomal dominant poly cystic kidney disease and also urinary tract infections. According to severity of secondary hyperparathyroidism, each patient being treated for secondary hyperparathyroidism was given oral active vitamin D3 (Rocaltrol), calcium carbonate, and Rena-Gel capsules of various doses. According to severity of anemia, patients underwent IV iron therapy with iron sucrose (Venofer) of various doses after each dialysis session. All the patients underwent treatments with 6 mg folic acid daily, 500 mg L-Carnitine daily, oral Vitamin B-complex tablet daily and also 2000U IV Eprex (recombinant human erythropoietin; (rHuEPO) after each routine dialysis session. Exclusion criteria were active or chronic infection and using NSAID or ACE inhibitor drugs.

### Laboratory methods

Complete blood count (hemoglobin & hematocrit level) analysis was performed using Sysmex-KX-21N Cell counter. Levels of serum iron, total iron binding capacity (TIBC) and serum ferritin, CRP and also levels of serum pre-dialysis creatinine, post- and pre-dialysis blood urea nitrogen (BUN), were measured using standard kits. For the efficacy of hemodialysis, the urea reduction rate (URR) was calculated from pre- and post-blood urea nitrogen (BUN) data (10). Duration and sessions of HD treatment were calculated from the patients' records. Each hemodialysis session was in duration of 4 hours.

### Ethical issues

(1) The research followed the tenets of the Declaration of Helsinki; (2) informed consent was obtained; (3) the research was approved by ethical committee of Shahrekord University of Medical Sciences.

### Statistical Analysis

For statistical analysis, data are expressed as the mean ± SD. Statistical correlations were assessed using partial correlation test. All statistical analyses were performed using SPSS 11.5 (SPSS Inc., Chicago, IL, USA). Statistical significance was determined at a *p*<0.05.

## Results

There were 36 patients overall (female=15, male=21). Mean patients' age was 53 (±15.8) years.

In this study, there was a significant inverse correlation between serum ferritin and hematocrit level (*r *=- 0.34, *p*=0.044) (adjusted for age). Also a significant positive correlation between serum CRP and serum iron (*r*=0.44, *p*=0.008) (adjusted for age) was observed. Moreover, in male HD patients, a significant positive correlation between serum CRP and serum ferritin (*r*=0.56, *p*=0.009) was registered.

## Discussion

The important finding of our study was a significant inverse correlation between serum ferritin and hematocrit level was seen. Also a significant positive correlation between serum CRP and serum iron was observed. Moreover, in male HD patients, a significant positive correlation between serum CRP and serum ferritin was registered. Anemia is a consistent finding in chronic renal disease, affecting up to 90% of patients. The central role of anemia in the development of cardiovascular dysfunction is now well-known (1-5). Anemia of HD can be managed relatively successfully by recombinant human erythropoietin. Iron administration plays a central role in enhancing anemia responsiveness to EPO. Serum ferritin concentration is a commonly used marker of iron status in maintenance dialysis patients (1-3). It was shown that a low serum ferritin concentration is a reliable indicator of iron deficiency among HD patients. However, a high serum ferritin may not be an optimal indicator of increased iron stores among dialysis patients, because it is an acute-phase reactant and its increase in dialysis patients may be based on the factor unrelated to iron stores such as inflammation (3-8). In dialysis patients, high CRP levels are associated with low hemoglobin levels and/or EPO resistance (11-13). In the study conducted by Sirken *et al*. it was found that the increased levels of CRP were associated with relative EPO resistance in dialysis patients (14). Our findings support the first direct evidence that inflammation, which is closely related to protein-energy malnutrition (3-8) in dialysis patients, might affect anemia toward its intensification. Our data support the evidence that inflammation may not have an effect on serum ferritin, unless there is enough iron stores in the body, so that serum ferritin is somewhat increased (2-7). Rogers *et al*. showed that IL-1ß induces ferritin gene expression by translational control of its mRNA. However, this inflammatory induction of ferritin synthesis is different from iron-dependent ferritin gene expression (9). They showed that this inflammatory regulation of ferritin requires the background presence of cellular iron (9). In other words, without adequate iron stores, serum ferritin is low and does not correlate with inflammation, but with enough iron, serum ferritin has the function of both iron and inflammation (2). Kalantar-Zadeh *et al*. in a study on of 82 HD patients, showed that serum ferritin concentrations were higher in malnourished patients (15).

## Conclusion

In summary, we can conclude that Malnutrition-inflammation complex syndrome may increase serum ferritin concentration apart from iron status, and needs more attention during iron therapy for the treatment of anemia in regular HD patients.

## Authors’ Contributions

HN defined the aims of research. HN prepared the paper. MRK edited the manuscript.

## Conflict of Interests

The authors declare that they have no conflict of interest.

## Ethical considerations

Ethical issues (including plagiarism, data fabrication, double publication) have been completely observed by the author.

## Funding/Support

This study was supported by a grant from Shahrekord University of Medical Sciences.
